# Low incidence of significant hydrogel spacer rectal wall infiltration: results from an experienced high-volume center

**DOI:** 10.3389/fonc.2025.1563015

**Published:** 2025-03-03

**Authors:** Sungmin Woo, Anton S. Becker, Aaron E. Katz, Angela Tong, Hebert A. Vargas, David J. Byun, Jonathan W. Lischalk, Jonathan A. Haas, Michael J. Zelefsky

**Affiliations:** ^1^ Department of Radiology, NYU Grossman School of Medicine, New York, NY, United States; ^2^ Department of Urology, NYU Grossman Long Island School of Medicine, Mineola, NY, United States; ^3^ Department of Radiation Oncology, NYU Grossman School of Medicine, New York, NY, United States; ^4^ Department of Radiation Oncology, Perlmutter Cancer Center at New York University Langone Hospital-Long Island, New York, NY, United States; ^5^ Department of Radiation Oncology, Perlmutter Cancer Center at New York University Langone Hospital-Long Island, Mineola, NY, United States

**Keywords:** hydrogel, magnetic resonance imaging, radiotherapy, spacer, rectal wall infiltration

## Abstract

**Objectives:**

To evaluate the incidence and degree of rectal wall infiltration (RWI) of spacer gel used during prostate radiotherapy among two practitioners experienced in using rectal spacers.

**Materials and methods:**

Consecutive patients with prostate cancer who received prostate radiotherapy after hydrogel rectal spacer insertion in August 2023–August 2024 by two experienced practitioners were retrospectively included. Post-implant magnetic resonance imaging examinations were evaluated by two radiologists for RWI: 0 (no abnormality), 1 (rectal wall edema), 2 (superficial RWI), and 3 (deep RWI). Scores 2–3 were considered positive for RWI and their location and degree of RWI (radial, longitudinal, and circumferential) were also categorized. Inter-reader agreement was assessed with Cohen’s Kappa.

**Results:**

215 men were included. Agreement was substantial between the radiologists for RWI scores (Kappa, 0.697; 95% confidence interval, 0.594-0.800). RWI scores were 0 in 80.5% (173/215), 1 in 7.9% (17/215), 2 in 10.7% (23/215), and, 3 in 0.9% (2/215) of the men. Altogether, RWI was present (scores 2–3) in 11.6% (25/215), most commonly in the mid-gland and apex with median radial, longitudinal, and circumferential involvement of 3.2 mm, 8.6 mm, and 11.5%. None of these patients demonstrated any significant rectal toxicity.

**Conclusion:**

RWI was very uncommon for experienced practitioners. The degree of RWI was focal and not associated with increased complications.

## Introduction

Hydrogel rectal spacer has been shown in three prospective randomized trials to be associated with reduced radiation dose of the rectum during prostate radiotherapy and improved tolerance of prostate radiotherapy with decreased acute and late treatment related toxicities (1–3). The increased space created by the instilled gel in the peri-rectal space effectively reduces the volume of the rectum exposed to the prescribed doses of radiotherapy. Acute rectal toxicity was noted to be improved in these studies, and in one study with longer follow-up (1), the incidence of grade 2 rectal toxicity at 2 years was reduced from 6% to 0%. While complications associated with the procedure have been uncommon, concerns have been raised regarding observations of rectal wall infiltration (RWI) of the gel and its uncertain association with subsequent rectal wall injury (4, 5). In an initial analysis reported by Fischer-Valluck et al. (6) based on the randomized Space-OAR trial, the incidence of RWI was noted to be 6%. However, on a subsequent analysis using a rectal classification grading system based upon depth of penetration of the gel into the rectal wall (7), the incidence of any RWI based on post-rectal spacer MRI assessments was noted to be 24%, yet significant RWI with partial penetration or deeper muscle infiltration was noted in 20% and 4%, respectively.

The causes of rectal wall infiltration have been hypothesized to be related to several factors. These may include inadvertent movement of the needle during gel deployment with injection into the rectal wall where the needle visualization may be obscured during ultrasound imaging, scarring in the peri-rectal space associated with weakened rectal muscle layer which may predispose to rectal penetration, pressure of injection in relation to the prostate level, or possibly too rigorous of an injection technique. A prior report demonstrated a higher incidence of RWI among practitioners with early experience (8), suggesting that this may be an important variable associated with better outcomes and lower incidence of RWI could be further mitigated with careful attention to technique during the procedure and frequency of the practitioner having performed the procedure.

In this report, we summarize the incidence and degree of RWI among two experienced practitioners with long term established experience and high-volume practices using SpaceOAR. In all patients the presence and degree of RWI was evaluated on post-spacer magnetic resonance imaging (MRI). For each case, two expert radiologists independently reviewed the MRIs and classified the RWI according to a previously published grading system (7). Our results are consistent with the notion that RWI is uncommon in experienced practitioners where careful technique is utilized.

## Materials and methods

### Patient cohort

This retrospective study was conducted at a single institution after approval from the institutional review board (BLINDED) in compliance to the Health Insurance Portability and Accountability Act and in accordance with the Declaration of Helsinki. The need for informed consent was waived due to the retrospective design of the study. The electronic medical records were searched to identify all consecutive patients with localized prostate cancer who received prostate radiotherapy after insertion of hydrogel rectal spacer between August 2023 and August 2024 by two practitioners specialized in prostate cancer treatment (BLINDED and BLINDED with more than 7 and 5 years, respectively, of experience in prostate radiotherapy using rectal spacers). Most patients were treated with stereotactic radiosurgery to prescription doses ranging from 35 Gy to 40 Gy in 5 fractions and their MRIs obtained 1 week after rectal spacer placement were used for treatment planning. In general, patients were injected with 10 cc of rectal spacer, although for patients with smaller prostates a smaller amount could be utilized. Patients received clinical follow up after treatment during the first year every 3 months and subsequently every 6 months.

### MRI evaluation for hydrogel rectal wall infiltration

Assessment of hydrogel rectal wall infiltration was done using MRI as the patients in this study underwent radiotherapy with MRI-based treatment planning. In addition, it has been shown that MRI is superior to CT for delineating the layers of the rectal wall or rectal involvement of various pelvic pathologies (9, 10).

MRI interpretations were done by two subspecialized genitourinary radiologists (BLINDED and BLINDED with more than 9 and 5 years of experience, respectively, in interpreting prostate MRI with and annual volume of >400 cases). The radiologists were aware patients had undergone radiotherapy to the prostate after insertion of rectal hydrogel spacer, but were otherwise blinded to the clinical and pathological data. MRI interpretations for RWI were first done independently, followed by a joint interpretation to reach a consensus for discrepancies.

**Figure 1:**
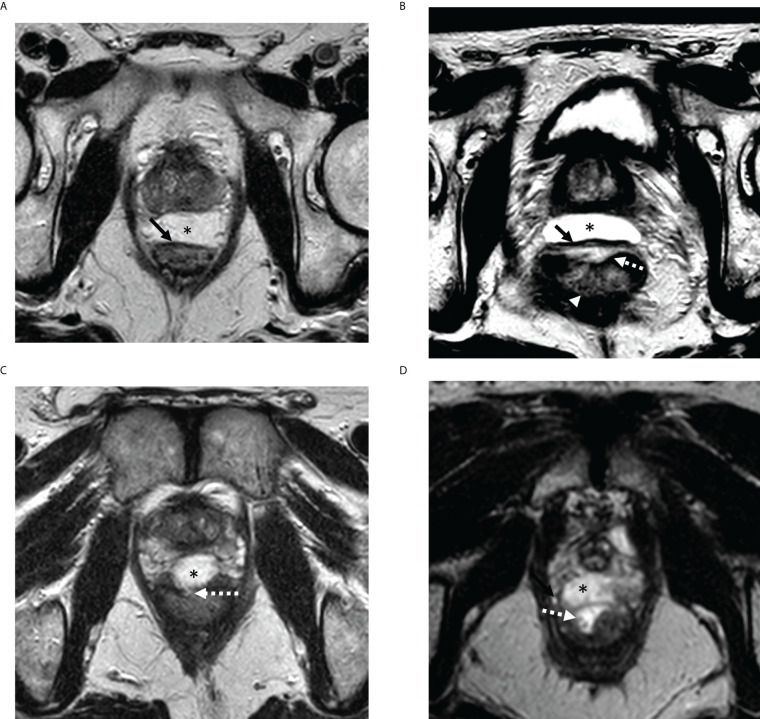
Representative MRI examples of rectal wall infiltration (RWI) scoring system on axial T2-weighted imaging. **(A)** RWI score 0: Hydrogel (*) is well circumscribed and outer wall of rectum is smooth (solid arrow) with no signal changes. **(B)** RWI score 1: Edema only appreciated in the anterior rectal wall (broken arrow) but outer rectal wall is smooth without breach (solid arrow). Note lack of edema in the posterior rectal wall (arrowhead) **(C)** RWI score 2 (Superficial RWI): Focal breach (broken arrow) is noted in outer rectal wall contiguous with the hydrogel (*). **(D)** RWI score 3 (Deep RWI): Hydrogel (*) extends to inner rectal wall and lumen (broken arrow). RWI, rectal wall infiltration.

The presence and degree of RWI was evaluated using multiplanar T2-weighted imaging (T2WI) in the axial, sagittal, and coronal planes, and were categorically scored from “0” to “3” according to a recently established Likert scoring system developed and subsequently validated from a multicenter randomized controlled trial (7, 11). In brief, scores of 0–3 were defined as the following (**Figure 1**): “0”, when no abnormal signal intensity (SI) was present in the rectal wall; “1”, when there was increased T2WI SI suggestive of edema within the rectal wall, but no breach of the hydrogel was seen through the rectal wall; “2”, when there was disruption of the outer muscularis propria layer of the rectal wall which appears typically hypointense on T2WI (i.e. “superficial” RWI); and “3”, when more inner layers were breached including the mucosal and submucosal layers which normally show mildly higher T2WI SI (i.e. “deep” RWI). Scores of 2–3 were considered positive for RWI and score of 0–1 were considered negative for RWI. Specifically, RWI score of 1 was thought to represent reactive changes to the hydrogel insertion procedure itself and not in itself a direct breach of the rectal wall from RWI.

**Figure 2:**
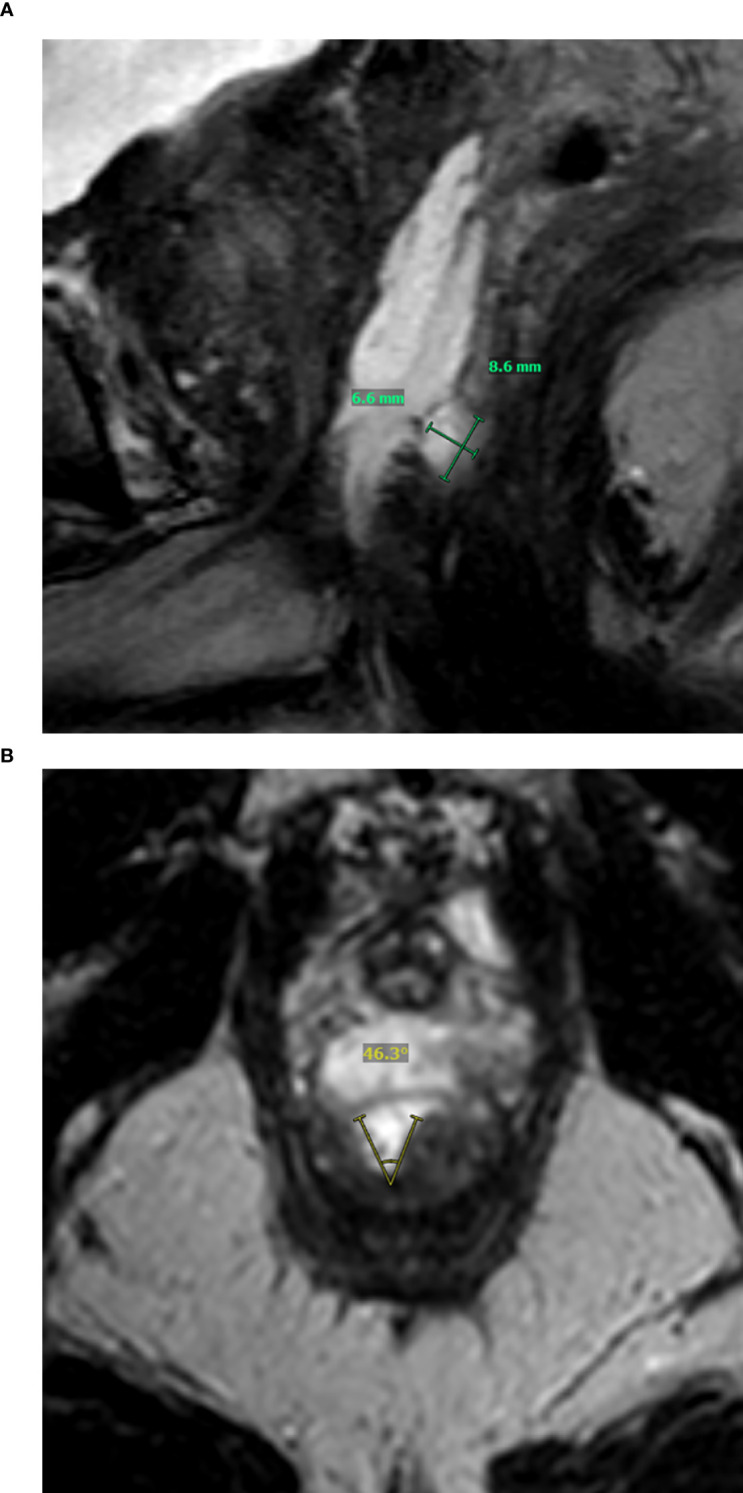
Representative example showing assessment of location and extent of rectal wall infiltration (RWI) on MRI. **(A)** Sagittal T2-weighted imaging shows deep RWI (score 3) at the apex with radial depth of 6.6 mm and longitudinal length of 8.6 mm. **(B)** Axial T2-weighted imaging shows that RWI involves 46.3°C (12.9%) of the rectal circumference. RWI, rectal wall infiltration.

For the patients that had either superficial or deep RWI (i.e., scores of 2–3), further analysis was done by the two radiologists in consensus to assess the extent of RWI as the following (**Figure 2**) (1): the anatomical level of RWI (e.g., base, mid-gland, and apex); (2) radial depth of RWI; (3) longitudinal length of RWI; and (4) circumferential percentage of involvement of RWI.

### Statistical analysis

Continuous variables were summarized as median and interquartile ranges and categorical variables as percentages and frequencies. Inter-reader agreement was assessed using the Cohen’s kappa statistic and the degree of agreement was categorized as the following: ≤0.20, as none to slight agreement; 0.21–0.40, fair agreement, 0.41–0.60, moderate agreement, 0.61–0.80, substantial agreement, and ≥0.81, almost perfect agreement (12). No other advanced statistical methods were used for this study. All statistical analyses were done using R version 4.3.0 (R Project for Statistical Computing, Vienna, Austria).

## Results

A total of 215 men were included in the analysis. There was substantial agreement between the two radiologists for RWI scoring with a Cohen’s kappa of 0.697 (95% confidence interval [CI], 0.594-0.800) and percentage agreement of 85.6% (184/215). The discrepancy matrix for RWI scores of the two radiologists are shown in **Table 1**. Of note, the majority of disagreements (60%) were between scores 0 and 1.

**Table 1:** Discrepancy matrix of RWI scores of two radiologists.

RWI score		Radiologist 1			
		0	1	2	3
Radiologist 2	0	160	0	2	0
	1	18	7	1	0
	2	2	3	16	1
	3	1	0	3	1

RWI, rectal wall infiltration.

Data are frequencies.

The RWI scores for each radiologist and that based on the consensus interpretation are provided in **Table 2**. The majority (80.5% [173/215]) of the men did not show any abnormal signal or breach of the rectal wall (score 0). A minority of the men demonstrated findings of rectal wall edema (score 1) or superficial RWI (score 2) (7.9% [17/215] and 10.7% [23/215], respectively). Deep RWI (score 3) was only seen rarely, in 0.9% (2/215) of the men. Overall, 11.6% (25/215) of the men were considered positive for RWI (score of 2–3). In these patients, RWI was seen most commonly in the mid-gland and apex with a median radial depth of 3.2 mm, median longitudinal length of 8.6 mm, and median circumferential involvement of 11.5% as summarized in **Table 3**.

**Table 2:** Distribution of RWI scores of two radiologists and by consensus interpretation.

RWI score	Definition	Radiologist 1	Radiologist 2	Consensus
0	No abnormal signal	181 (84.2)	162 (75.3)	173 (80.5)
1	Edema only	10 (4.7)	26 (12.1)	17 (7.9)
2	Superficial RWI	22 (10.2)	24 (11.2)	23 (10.7)
3	Deep RWI	2 (0.9)	5 (2.3)	2 (0.9)

RWI, rectal wall infiltration.

Data are frequencies with the percentages in parentheses.

**Table 3:** Characteristics of RWI.

Characteristics	Data
Level of RWI	
Base	2.0 (8.0)
Mid-gland	13.0 (52.0)
Apex	10.0 (40.0)
Radial depth (mm)	3.2 (2.7–4.3)
Longitudinal length (mm)	8.6 (7.4–10.6)
Circumferential involvement (%)	11.5 (7.6–15.0)

RWI, rectal wall infiltration.

Data are frequencies with the percentages in parentheses or medians with interquartile ranges in parentheses.

Among the patients with RWI scores of 2 and 3, one patient with grade 2 RWI developed prostatitis 6 months after treatment which subsequently resolved with antibiotic therapy and presumed to be unrelated to the spacer placement; otherwise, no patient developed significant grade 2 or higher rectal toxicity associated with the rectal spacer placement. Due to the low incidence of RWI, it was not feasible to assess the relationship with volume of injected rectal spacer. No patients had delay in implementation or initiation of their radiotherapy treatments related to RWI.

## Discussion

The incidence of MRI-assessed RWI is very rare among practitioners who frequently perform rectal hydrogel spacer placement, as clearly demonstrated in our findings. Superficial RWI incidence was approximately 10% and deep (scored as grade 3) RWI was <1%. Even among those with some degree of RWI, most cases were noted to be only subcentimeter.

Attention to detail related to the technical aspects of the procedure is critical for reducing the incidence of RWI during hydrogel rectal spacer placement. Several approaches in our experience that have helped reduce the incidence of RWI include ensuring that the operator visualizes the needle at all times during the procedure viewing on the axial and sagittal images. Optimally, the injection of the hydrogel should be deployed in the sagittal plane and care should be taken during injection to avoid significant movement of the needle. In addition, the injection of the gel should be directed into the fluid bubble created by the hydro-dissection, and the needle tip should be steered away from the rectal wall. Finally, the injection should be deployed without any excessive force, and if resistance is encountered, consideration should be given for aborting the cases as there may be, in such cases too much fibrosis within the peri-rectal space.

In our patient population, even among those with superficial or deep RWI, we did not observe an association with subsequent rectal toxicity. These findings are consistent with recently reported observations from Grossman et al. (7) where 20% and 4% of patients experienced grade 2 (superficial) and grade 3 (deep) RWI, there were no occurrences of rectal toxicity in these latter patients, and no differences in acute or late toxicity were detected for any of the RWI grades compared to patients who had no imaging evidence of RWI after hydrogel spacer placement.

The observation that RWI was not directly associated with rectal toxicity suggests that the mere penetration of the gel, especially when the infiltration is focal in nature, does not necessarily result in any significant compromise in the integrity of the rectal wall. Complications, when they manifest, may be more likely observed with rectal penetration associated with a greater span involvement of the rectum. The risk may be further elevated in the setting of an underlying infection where compromise of the integrity of the rectal wall could lead to the development of a severe ulcer. If this is correct, cases where RWI of significance is noted may be helped with antibiotic coverage. In fact, our clinical practice has been to delay radiotherapy in such cases until the hydrogel naturally dissolves. Larger studies will be necessary to confirm these findings.

There are some limitations of this study. First, there is inherent bias related to the retrospective design. Second, it was performed at a single institution and therefore multicenter studies may be needed for validation. Finally, as the results are from experienced practitioners with high-volume practices using SpaceOAR, caution is needed in applying the results to those with less experience.

In conclusion, RWI is very uncommon in the hands of experienced practitioners who have a high volume of performing such procedures. Yet, while these procedures are considered low risk for toxicity, they nevertheless should be done in a high volume setting with experienced practitioners where the incidence of RWI would be low as observed. The incidence of complications was minimal but this could be related to the overall low incidence of high grade RWI, and the fact that even among patients who had some degree of RWI it was quite focal and not spanning a large involvement of the rectal wall. Nevertheless, RWI should be avoided or minimized and attention to technical details in the deployment of the rectal spacer is critical.

## Data Availability

The raw data supporting the conclusions of this article will be made available by the authors, without undue reservation.
